# Supplementary material for: Predicting gene expression using DNA methylation in three human populations
Source: PeerJ. 2019 May 1;7:e6757. doi: 10.7717/peerj.6757 (PMC6500370; doi:10.7717/peerj.6757)
Supplement: Supplemental Information 1 — Additional data and results for supplementary table 1 and supplementary figure 1-4. [file peerj-07-6757-s001.pdf]

Datasets	CpG Probe Type	CpG Probes		Genes	
		w/LASSO model	w/o LASSO model	w/LASSO model	w/o LASSO model
Adipose	Methyl Probes	149152	276463	8040	16800
	S&C probes	46563	114609	5645	17018
	All probes	211020	373691	8864	17872
PBMC	Methyl Probes	73553	404434	4252	24972
	S&C probes	28345	152568	3435	22808
	All probes	107484	541550	5064	25966
LCL	Methyl Probes	143599	426146	7514	25432
	S&C probes	40559	150712	5241	22987
	All probes	173832	507339	7498	24178

Supplementary Table 1. Numbers of CpG probes and genes that have valid LASSO models in the three datasets. LASSO regression models were fitted using three different categories of CpG probes separately, DNA methylation (Methyl) probes, SNP or cross-hybridization (S&C) probes, and all probes. 'w/LASSO model' indicates the number of CpG probes or genes that have valid LASSO models; 'w/o LASSO model' shows the number of CpG probes or genes failed to generate a valid LASSO model, which indicates lack of predictive information in DNA methylation.

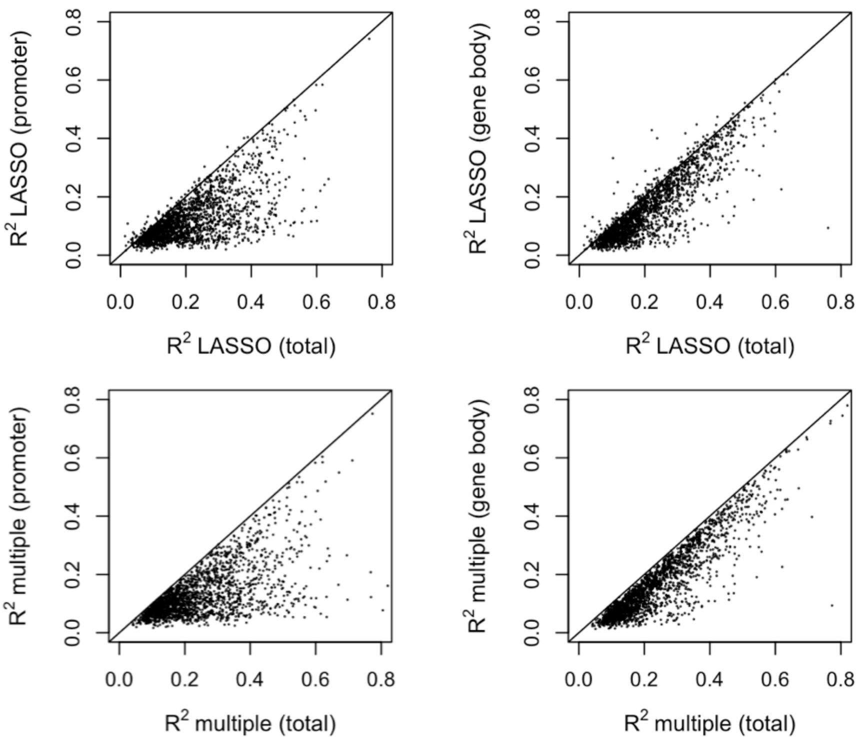

Supplementary Figure 1. Separating CpG sites into promoter region and gene body does not improve LASSO prediction or multiple prediction. Gene region CpG sites were grouped into promoter region and gene body region based on their location relative to gene model. The two groups were modeled separately using LASSO regression. Their prediction power is mostly lower than the combined. "R^2 LASSO (total)", R^2 from LASSO regressions model using all methylation probes of the corresponding gene; "R^2 LASSO (promoter)", R^2 from LASSO regressions model using methylation probes located in the promoter region; "R^2 LASSO (body)", R^2 from LASSO regressions model using methylation probes located in body region. "R^2 multiple (total)", R squared of multiple regressions model using all methylation probes of the corresponding gene. "R^2 multiple (promoter)", R^2 from LASSO regressions model using methylation probes located in promoter region. "R^2 multiple (body)", R^2 from multiple regressions model using methylation probes located in body region.CpG probes annotations were downloaded from https://support.illumina.com, Infinium HumanMethylation450 BeadChip. These results are from LCL dataset.

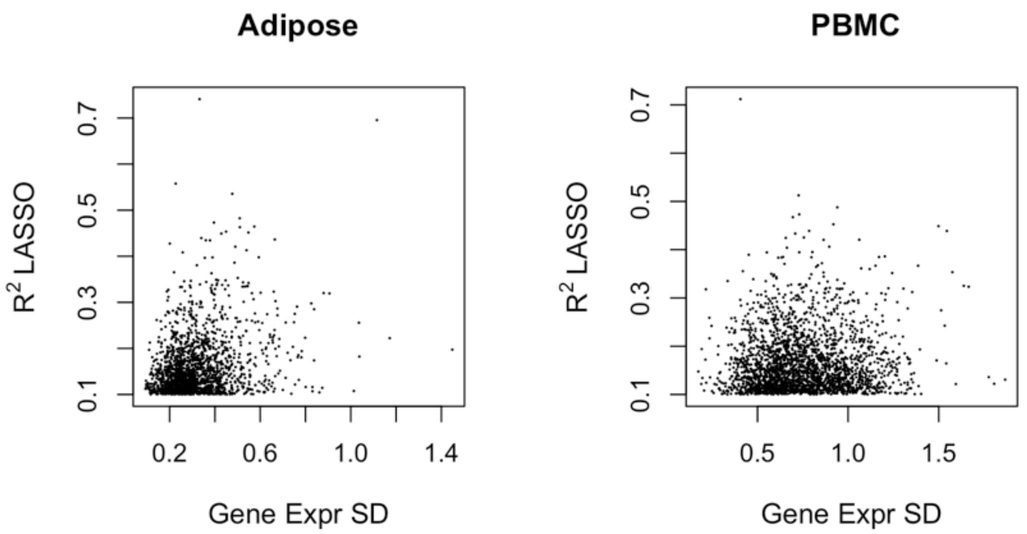

Supplementary Figure 2. R^2 from LASSO regression is not highly associated with Gene expression variation. R^2 values were generated from methylation CpGs. Genes with LASSO R^2 larger than 0.1 are shown. Gene Expr SD: Gene expression standard deviation.

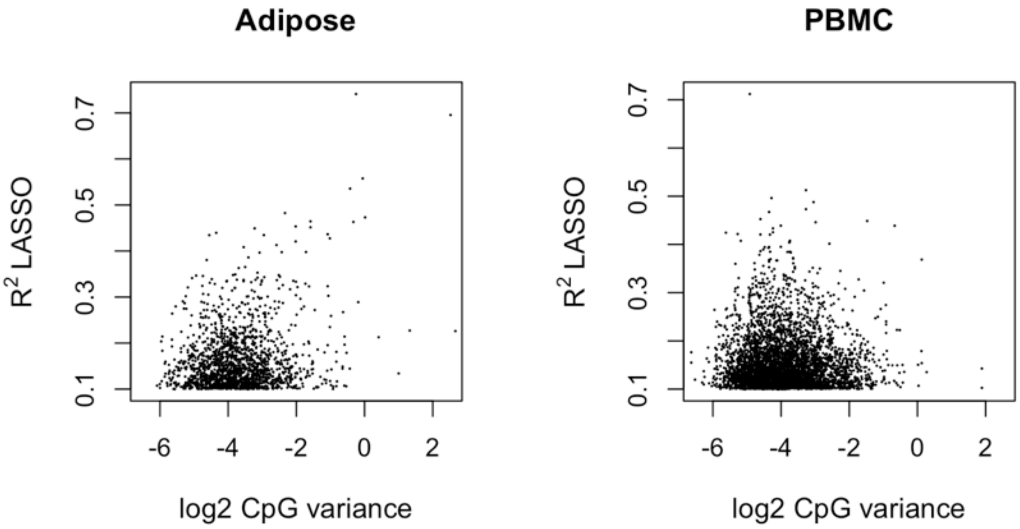

Supplementary Figure 3. R^2 from LASSO regression is not associated with CpG methylation variation. CpG methylation variance is in log2 transformed and is calculated from the methylation CpG site that has the maximum R^2 in single regression at each gene. Genes with LASSO R^2 larger than 0.1 are shown.

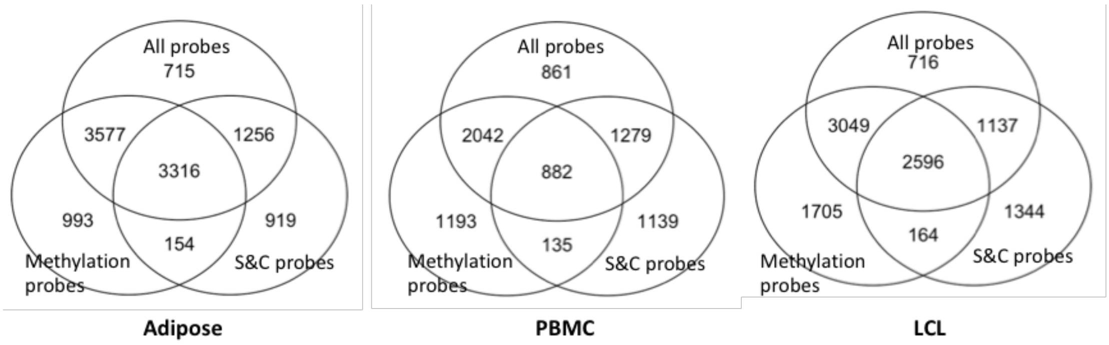

Supplementary Figure 4. Overlap of genes with valid LASSO models from different types of CpG probes. All, all CpG probes; Methylation, methylation CpG probes; S&C, SNP and cross hybridization probes.
